# Retinoblastoma Loss Modulates DNA Damage Response Favoring Tumor Progression

**DOI:** 10.1371/journal.pone.0003632

**Published:** 2008-11-05

**Authors:** Marcos Seoane, Pablo Iglesias, Teresa Gonzalez, Fernando Dominguez, Maximo Fraga, Carlos Aliste, Jeronimo Forteza, Jose A. Costoya

**Affiliations:** 1 Molecular Oncology Lab, Departamento de Fisioloxia, Facultade de Medicina, Universidade de Santiago de Compostela, Santiago de Compostela, Spain; 2 Fundacion Galega de Medicina Xenomica, Servicio Galego de Saude, Santiago de Compostela, Spain; 3 Departamento de Anatomia Patoloxica e Ciencias Forenses, Universidade de Santiago de Compostela, Santiago de Compostela, Spain; Cleveland Clinic, United States of America

## Abstract

Senescence is one of the main barriers against tumor progression. Oncogenic signals in primary cells result in oncogene-induced senescence (OIS), crucial for protection against cancer development. It has been described in premalignant lesions that OIS requires DNA damage response (DDR) activation, safeguard of the integrity of the genome. Here we demonstrate how the cellular mechanisms involved in oncogenic transformation in a model of glioma uncouple OIS and DDR. We use this tumor type as a paradigm of oncogenic transformation. In human gliomas most of the genetic alterations that have been previously identified result in abnormal activation of cell growth signaling pathways and deregulation of cell cycle, features recapitulated in our model by oncogenic Ras expression and retinoblastoma (*Rb*) inactivation respectively. In this scenario, the absence of pRb confers a proliferative advantage and activates DDR to a greater extent in a DNA lesion-independent fashion than cells that express only HRas^V12^. Moreover, *Rb* loss inactivates the stress kinase DDR-associated p38MAPK by specific Wip1-dependent dephosphorylation. Thus, *Rb* loss acts as a switch mediating the transition between premalignant lesions and cancer through DDR modulation. These findings may have important implications for the understanding the biology of gliomas and anticipate a new target, Wip1 phosphatase, for novel therapeutic strategies.

## Introduction

Cell proliferation is dependent on the appropriate transmission of growth signals from the cell membrane to the nucleus. Ras family proteins function as a membrane-associated biologic switch that relays signals from ligand-stimulated receptors to cytoplasmatic downstream effectors proteins like phosphoinositide 3-kinase (PI3K) and Raf-MEK1/2. These protein-kinases are responsible of phosphorylation of transcription factors, which in last instance contribute to proliferation, differentiation and cell survival. The importance of the Ras/Raf/MEK/ERK pathway in growth control is further supported by the fact that hyperactivation of this pathway is usually found associated with many human tumors such as gliomas.

Gliomas are the most common primary tumors in the brain and are divided into four clinical grades on the basis of their histology and prognosis [1]. Several gene expression alterations and chromosomal abnormalities are commonly found in gliomas, and in some cases these mutations correlate with clinical grade. One common alteration in gliomas of all grades is the overproduction of growth factors such as FGF2, EGFR and PDGF19, and their receptors. Interestingly, although mutations in Ras are not found in gliomas, overexpression and/or gain-of-function mutations in several growth factor receptors increase Ras activity [2]–[4], activating downstream effectors like Raf/MEK/ERK and PI3K/AKT pathways [5]. Indeed, several mouse models of glioma have causally implicated activated HRas and KRas in glioma formation [6]–[8].

Deregulation of signal transduction pathways mutations is found both in low- and high-grade gliomas, whereas other mutations are predominantly found in one or another group of gliomas. Thus, nearly all WHO grade III and IV (GBM) show a disruption of cell cycle due to alterations in one out of three genetic loci governing G1 arrest such as: *CDK4*, *INK4a–ARF* or *Rb*. Over half of high-grade human gliomas lack a functional *INK4a–ARF* locus [9], [10] and hence can produce neither p16^INK4a^ nor p19^ARF^, the two proteins encoded by this locus [11]. Most of the remaining gliomas either demonstrate a great amplification of the *CDK4* locus or lack the *Rb* gene [12]–[14] conjointly, in some cases, with p53 alterations [15].

Curiously, in human and rodent cells, oncogenic signals result in a permanent G_1_ arrest known as OIS [16], [17]. Although diverse stimuli can induce a senescence response, they appear to converge on p16^INK4a^/pRb and/or p19^ARF^/p53 pathways that establish and maintain the senescence growth arrest. In fact, increased expression levels of p53, p19^ARF^ and p16^INK4a^ have been found in murine cells undergoing OIS, as well as in some human premalignant neoplastic lesions [18]–[21].

Several oncogenes like Ras induce DNA replication stress, which leads to activation of the DNA damage response (DDR). This response includes phosphorylation of kinases such as ATM and Chk2, histone H2AX and p53 [19], [20], [22], which ultimately force the cell to undergo oncogene-induced senescence (OIS). Like apoptosis, OIS is a tumor-suppressing defense mechanism that must be compromised for tumorigenesis to occur.

In the same way, recent studies have revealed a major role of the p38MAPK pathway in OIS caused by oncogenic Ras [23], [24]. Although, part of the tumor-suppressive function of p38MAPK is attributed to its ability to mediate apoptosis as a result of ROS accumulation induced by activated oncogenes [25].

Here, we have developed a new model for gliomagenesis in hopes of understanding these patterns and discerning the contributions made to tumor formation. Similar approaches have been used before by other groups to recreate the behavior of glioma cells introducing in astrocytes some of the genetic lesions mentioned above [26]–[28]. We demonstrate that astrocytes are resistant to Ras-induced senescence in presence of high levels of ROS and DNA damage despite DDR activation. Moreover, in this context, loss of *Rb* locus favors the progression of low-grade gliomas to higher-grade tumors by conferring a double selective advantage to the tumor. First, deregulation of the pRb-E2F1 pathway is able to stimulate DDR activation through activation of ATM to phosphorylate p53 without increasing DNA double-strand breaks (DSBs) or ROS production [29]. This preferential activation of the DNA damage response increases DNA repair capacity that is essential for tumor cell survival. Finally, loss of *Rb* locus negatively regulates p38MAPK phosphorylation. Down-regulation of the p38MAPK signaling pathway is achieved through E2F-induced up-regulation of Wip1, a specific phosphatase that dephosphorylates and inactivates p38MAPK [30], also favoring the progression to astrocyte transformation.

## Results

Considering that upregulation of growth factor signaling pathways and the loss of cell cycle regulators are common features in glioblastoma multiforme [31], we address the role of HRas^V12^ and/or *Rb* loss in conditional *Rb* murine astrocytes to explain the tumor initiation and maintenance. To this purpose, conditional *Rb* mutant mouse astrocytes were infected by retrovirus encoding mutant *Ras* allele (HRas^V12^) and the recombinase Cre that targets the *loxP* sequences flanking exon 19 of the *Rb* gene [32]. Thus, we managed to mimic the characteristic upregulation of EGFR and FGFR signaling pathways by introducing a constitutively activated isoform of Ras, and the cell cycle deregulation by genetically inactivating the pocket protein Rb.

However, previous studies have reported that primary MEFs enter senescence upon oncogenic stress induced by ectopic expression of the oncogenic HRas^V12^ [17], [33]. Also, it has been reported that the absence of pRb enables Ras activation modulating differentiation in MEFs [34]. Accordingly, we carried out several infection assays on MEFs where we observed that all experimental groups enter senescence after HRas^V12^ expression (data not shown), as others reported.

**Figure 1:**
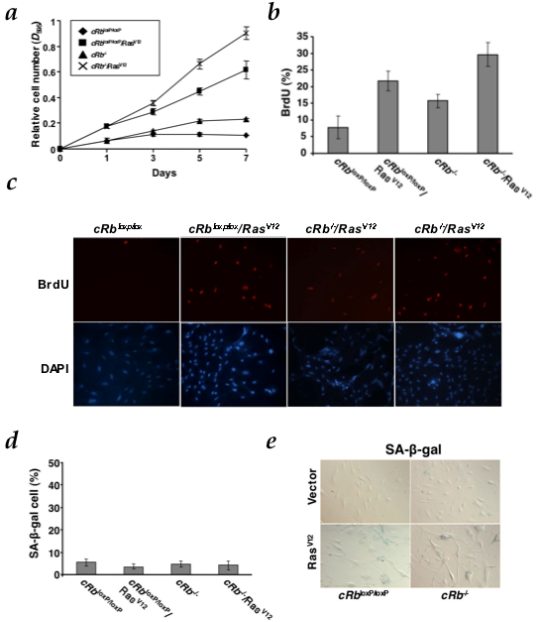
Mouse astrocytes are defective for HRasv12-induced cell cycle arrest. a, Growth curve analysis of early-passage c*Rb* ^loxP/loxP^ conditional astrocytes co-infected with PIG/pBABE (vector), PIG/pBABE-HRas^V12^, PIG-Cre/pBABE and PIG-Cre/pBABE-HRas^V12^ retroviral vectors. After infection, cells were plated in triplicate and the cells were fixed on the indicated days for subsequent staining with crystal violet. Each time point represents the mean±s.d. of total cumulative cell number from at least three independent experiments. b, Measurement of the proliferation of astrocytes by BrdU incorporation assay (panel c). c, Cells were labeled with BrdU for 5 h on day 5 after puromycin selection. d, Senescence assays in c*Rb^loxP/loxP^* and c*Rb^−/−^*. The y-axis represents the percentage of SA-β-galactosidase-positive cells (mean and s.d.) from at least three independent experiments. e, A representative result of three independent experiments is shown. Photographs are at the same magnification.

Unexpectedly, we observed that the astrocytes infected with PIG-Cre/pBABE-HRas^V12^ retroviral vector (hereafter c*Rb* ^−/−^/Ras^V12^) showed after one week an increased proliferation rate when compared with the remaining groups. Astrocytes infected with PIG-Cre/pBABE (hereafter c*Rb* ^−/−^) and PIG/pBABE (hereafter c*Rb* ^loxP/loxP^) were used as control groups. To our surprise, astrocytes infected with PIG/pBABE-HRas^V12^ (hereafter c*Rb* ^loxP/loxP^/Ras^V12^) also displayed a dramatic increment when compared with the control group, although at a less extent than c*Rb* ^loxP/loxP^/Ras^V12^ astrocytes. Proliferative differences between astrocytic populations can be easily spotted in Figure 1A. To further support these data, we carried out a BrdU incorporation assay on all of the experimental groups, as shown in Figures 1B and 1C, that confirmed our initial observation.

These data is compatible with the observation that both c*Rb* ^loxP/loxP^/Ras^V12^ and c*Rb* ^−/−^/Ras^V12^ groups did not show SA-β-Gal positive staining (Figure 1E), either in the presence or absence of pRb. Thus, our findings suggest that oncogenic Ras alone, regardless of pRb status, is sufficient to increase proliferation in astrocytes and at the same time, circumvent the oncogene-induced senescence seen in MEFs. Furthermore, *Rb* loss cooperates with oncogenic Ras enhancing the growth rate but without inducing senescence (Figures 1D and 1E) or apoptosis in astrocytes (data not shown), as others previously reported in other cellular systems [35].

**Figure 2:**
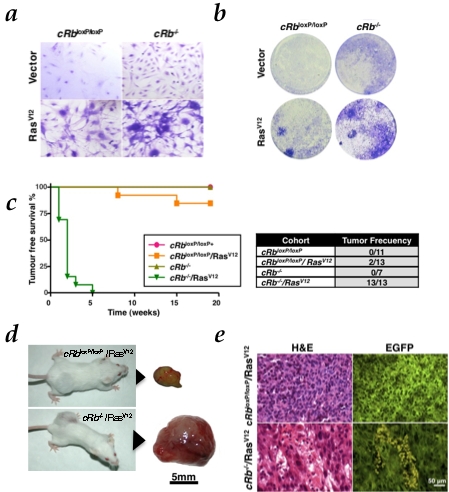
HRas^V12^ in the absence of *Rb* induces malignant transformation both *in vitro* and *in vivo*. a, Ability to form foci, representative pictures of wells stained with crystal violet (day 7). b, Representative pictures of wells stained on the indicated days of culture showing the morphological change observed in each group are also shown. c, Tumor-free survival curve (Kaplan-Meier plot) for tumor formation in SCID mice. Tumor was found in c*Rb* ^−/−^/Ras^V12^ cohort (P<0.0001) d, Photographs of SCID mice after subcutaneous injection of c*Rb* ^loxP/loxP^/Ras^V12^ and c*Rb* ^−/−^/Ras^V12^ astrocytes at eleven and two weeks respectively. Actual sizes of tumors after biopsy. e. Low-grade gliomas (c*Rb* ^loxP/loxP^/Ras^V12^; H&E, upper left) showed a rather monomorphic appearance, with polygonal or rounded cell shape and smaller rounded nuclei without prominent nucleoli. Note the absence of mitosis and necrosis. In contrast, high-grade gliomas (*cRb^−/−^*/Ras^V12^; H&E, lower left) displayed cellular pleomorphism, with large, fusiform and irregular nuclei that frequently exhibit mitotic figures (upper left corner) and necrotic foci (lower right corner). Co-expression of EGFP permits the visualization of the previously modified astrocytes.

Unlike normal primary astrocytes, flattened and non-refractile cells, Ras-expressing astrocytes showed noticeable morphological changes and loss of contact inhibition, and although flat cells were also present, the population was heterogeneous and large displaying at the same time a refractile cytoplasm with thin and long projections (Figure 2A). It is also revealing the fact that although *Rb*-deficient astrocytes displayed a marked proliferative increase its morphology did not appear significantly changed. On the contrary, Ras-expressing astrocytes seemed to undergo dramatic morphological alterations suggesting a transformed phenotype. We then wondered if these astrocytes would also exhibit foci growth, which is a characteristic feature of transformed cells. As Figure 2B shows, HRas^V12^ seems to induce foci growth as seen in c*Rb* ^loxP/loxP^/Ras^V12^ and c*Rb* ^−/−^/Ras^V12^ cells, whereas the remaining groups do not show significant foci formation.

To further support the hypothesis concerning the transformed status of these cells, we injected subcutaneously all the experimental groups in SCID mice. c*Rb* ^loxP/loxP^/Ras^V12^ astrocytes formed detectable tumors (N = 2/13; p = NS) within 8–15 weeks whereas tumors formed by c*Rb* ^−/−^/Ras^V12^ (N = 13/13; p<0.0001) appeared in-between the first and second week. No tumors were observed in mice injected with c*Rb* ^loxP/loxP^ (N = 0/11) or c*Rb* ^−/−^ (N = 0/7) astrocytes (Figures 2C and 2D). Together, our data suggest that HRas^V12^ and *Rb* loss promote the transformation of those cells. However, HRas^V12^ alone is also able to induce tumor growth in a minor extent and with a long latency, suggesting that in this case a second oncogenic event is needed to trigger tumorigenesis.

Histopathological examinations of the tumors generated from c*Rb* ^−/−^/Ras^V12^ cells showed a high degree of vascularization (Figure 2E) and necrotic regions surrounding the tumoral core, caused by the chaotic cell growth observed in the tumoral morphology resembling to those observed in human GBMs. However, the tumors observed in mice injected with c*Rb* ^loxP/loxP^/Ras^V12^ astrocytes bore a strong similarity with human low-grade gliomas, showing moderate cellularity and few mitosis (Figure 2E). Therefore, although in other cell systems like MEFs, [17], [21], [33] it seems to be indispensable the loss of at least one tumor suppressor to make cells more susceptible to Ras-induced hyper-replication effects. In primary astrocytes, HRas^V12^ oncogenic activity alone is sufficient to unleash cellular hyper-proliferation but not *in vivo* tumor formation. Then, *Rb* inactivation, besides enhancing cellular growth in HRas^V12^-expressing astrocytes (Figures 1A, 1B and 1C), also confers a higher aggressiveness to the tumor (Figures 2C and 2D).

**Figure 3:**
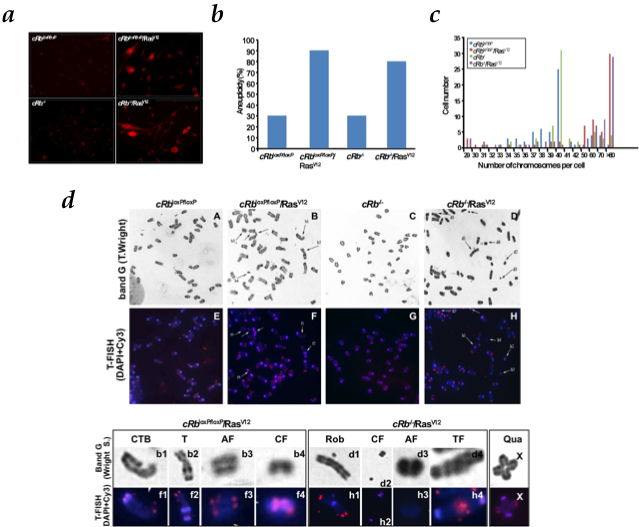
HRas^V12^-dependent production of reactive oxygen species (ROS) and associated chromosomal instability. a, cells were assayed for dihydroethidium (DHE) fluorescence, indicative of ROS production and visualized by fluorescent microscopy. Under identical imaging conditions, DHE oxidation was significantly increased in c*Rb^loxP/loxP^*/Ras^V12^ and c*Rb^−/−^*/Ras^V12^ astrocytes. b, *cRb^−/−^*/Ras^V12^ and *cRb^loxP/loxP^*/Ras^V12^ cells are significantly more aneuploid than c*Rb* ^loxP/loxP^ and c*Rb* ^−/−^ cells. c, Absolute chromosome numbers of c*Rb* ^loxP/loxP^, c*Rb* ^loxP/loxP^/Ras^V12^, c*Rb* ^−/−^ and c*Rb* ^−/−^/Ras^V12^ cells, showing the presence of diploid and tetraploid populations. Representative data of at least three independent experiments. d, Band G (Wright S) and telomere fluorescence in situ hybridization (T-Fish) was performed using a Cy3-labeled peptide nucleic acid (PNA) telomeric probe. Representative metaphase spreads from c*Rb* ^loxP/loxP^ and *cRb* ^−/−^, partial representative metaphase spreads from c*Rb* ^loxP/loxP^/Ras^V12^ and c*Rb^−/−^*/Ras^V12^ stained with Wright and hybridized with Cy3 telomeric probe are shown. The regions of metaphase spreads from c*Rb* ^loxP/loxP^/Ras^V12^ and c*Rb* ^−/−^/Ras^V12^ are magnified in the lower panel to show centromere fragments (CF), acentric fragment (AF), chromatide type break (CTB), telomeric fusion (TF), robertsonian translocation (Rob) and other type of translocation (T). X indicates quadrivalent (Qua) found in c*Rb* ^loxP/loxP^/Ras^V12^.

As we previously described, although astrocytes harboring HRas^V12^ did not enter senescence we wanted to confirm whether DNA damage provoked by DNA replication stress, and usually associated with oncogene-induced senescence, was present. On the other hand, HRas^V12^ is also known to cause an increase in reactive oxygen species (ROS) production [36] as a result of replication stress. ROS has been traditionally considered as a toxic by-product of cellular metabolism, but it has been appreciated that they are actively involved in oncogenic signaling in cellular transformation and cancer. Increased intracellular levels of ROS have also been reported to mediate some biological effects of oncogenic HRas^V12^, such as the onset of premature senescence in primary cells, the generation of genomic instability [37], and malignant transformation [38]. Furthermore, high levels of ROS have been detected in several human cancer cell lines as well as in human tumors from different tissues. Taken together these reports, we wanted to analyze in astrocytes the relationship between oxidative stress and glioma. We observed similar oncogene-induced ROS accumulation in c*Rb* ^loxP/loxP^/Ras^V12^ and c*Rb* ^−/−^/Ras^V12^ astrocytes, but not in those that had lost only *Rb* (Figure 3A). Likewise, we found signs of chromosomal instability (Figure 3B–D) in the groups that expressed HRas^V12^ and displayed ROS accumulation. One of the most remarkable features of c*Rb* ^loxP/loxP^/Ras^V12^ and c*Rb* ^−/−^/Ras^V12^ astrocytes is the abundance of centromeric instability manifested as centromere fragments (CF) (Figure 3D and Figure S1). We also found Robertsonian centromere fusion (Rob) in c*Rb* ^loxP/loxP^/Ras^V12^ and c*Rb* ^−/−^/Ras^V12^ astrocytes but not in c*Rb* ^−/−^ and wildtype astrocytes (Figure S1). In addition, there were numerical chromosome abnormalities in the experimental groups that expressed oncogenic Ras. These astrocytes presented >80 chromosomes per cell, doubling the chromosome number found in control cells (Figure 3B and 3C). Hence, these numerical and structural chromosome abnormalities show that activated Ras is sufficient to induce chromosomal instability in the absence of other signals, suggesting that Ras-induced chromosomal instability arises as consequence of ROS accumulation and DNA replication stress. In addition, there is not a significant difference between c*Rb* ^loxP/loxP^/Ras^V12^ and c*Rb* ^−/−^/Ras^V12^ astrocytes implying that the absence of pRb has not effect on DNA lesions, either quantitative or qualitative.

**Figure 4:**
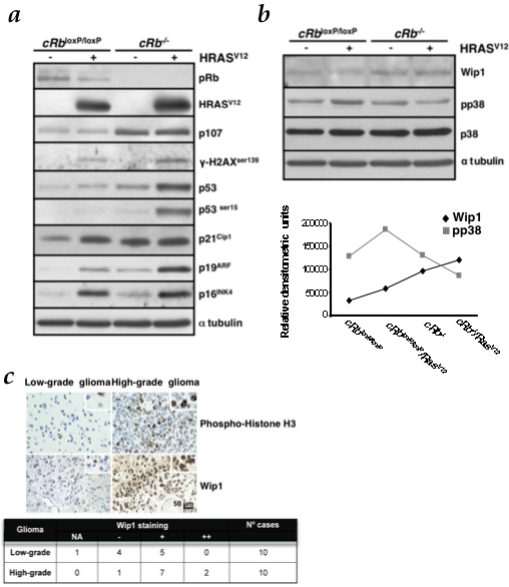
*Rb loss* enhances DNA damage response (DDR) induced by oncogenic Ras and reduces the p-p38MAPK levels by upregulation of Wip1. a, HRas^V12^ is able to induce DDR markers expression, such as p16^INK4a^, p21^Cip1^ and p19^ARF^, p53 and p53^ser15^ and γ-H2AX^ser139^. *Rb* loss, in the presence of oncogenic Ras, increases this response. Immunoblot analysis was performed on astrocytes lysates prepared at day 6 after co-infection and selection with puromycin. b, Immunoblot analysis of p-p38MAPK and Wip1 levels was performed on cell lysates prepared at day 5 from co-infection and selection with puromycin. Densitometric analysis (in relative densitometric units) of Wip1 and p-p38MAPK protein levels. c, TMA analysis of Wip1 expression in glioma clinical samples. Representative pictures of samples are shown. The table shows median values. Wip1 immunoreactivity intensity was assigned according to the following scale: NA, non-assessable cases; −, less than 10% of neoplastic cells displayed immunoreactivity; +, 11–29% of neoplastic cells displayed immunoreactivity; ++, 30% or greater percentage of neoplastic cells displayed immunoreactivity. Phospho-histone H3 was used as a proliferation marker. Scale bars, 50 µm.

These observations suggest that the presence of DNA lesions may trigger DDR activation. Although the mechanisms that generate DNA damage put in place by different oncogenic events are unclear presently, Ras activation induces not only ROS-induced DNA damage but also re-replication, an event known to cause DDR activation [21]. DDR is likely to be related to premalignant neoplastic lesions in human [18], [39]–[41], and has been linked to increased expression levels of p16^INK4A^ and p19^ARF^ [18], which appear mutated in a vast proportion of GBMs [31]. Consequently, and considering that these cells were proliferating, we expected these DDR checkpoints to appear down-regulated. Surprisingly, we observed a rise of p16^INK4A^, p19^ARF^ and p21^CIP1^ expression levels in HRas^V12^-expressing astrocytes despite of the lack of senescence induction. Accordingly, detection of p-p53^Ser15^ and phosphorylated histone γ-H2AX in HRas^V12^ expressing cells suggested DNA damage induced by the Ras-driven DNA hyper-replication (Figure 4A). Moreover, protein levels and their phosphorylation status were significantly higher in c*Rb* ^−/−^/Ras^V12^ astrocytes than c*Rb* ^loxP/loxP^/Ras^V12^ indicating that c*Rb* ^−/−^/Ras^V12^ cells showed higher checkpoint activation (Figure 4A).

This difference can be explained by the fact that *Rb* inactivation is able to trigger this response in a DNA damage-independent manner. Recently, it has been shown that DNA damage-resistant glioma cells show preferential activation of DDR contributing to an increased survival of these cells [42]. This response may be initiated by the recruitment of repair factors to the site of DNA damage to form a multiprotein repair complex. The activation of this response does not require DNA lesions [43], [44], which may be readily repaired in *Rb*-deficient cells due to the elevated levels of repair factors activated in these responses. In fact, loss of pRb may deregulate cell cycle checkpoints, allowing the propagation of deleterious mutations, such as the ones produced by Ras-induced hyper-replication to promote tumor progression [29], [45]. Interestingly, several studies have reported the instrumental role of p107 in compensating the *Rb* loss, thus preventing the cell to undergo transformation [33], [46]. However, up-regulation of this pocket protein due to *Rb* inactivation apparently has a null effect on Ras-expressing astrocytes (Figures 4A and S2).

Also, p38MAPK has been reported to function downstream DNA damage sensors such as ATM and ATR after genotoxic insult [47]. Besides that, the p38MAPK pathway is known to become activated upon cellular stress, blocking proliferation or promoting apoptosis [23]–[25], [48], [49]. p38MAPK mediates multiple cellular processes but apparently its role in stress response is one of the key factors in Ras-induced senescence. Thus, p38MAPK acts as a sensor of oxidative stress during the initiation of tumorigenesis and regulates negatively malignant transformation induced by oncogenic Ras [25]. Given that, we wanted to know how p38MAPK is regulated in our model.

Therefore, we next investigated whether p38MAPK phosphorylation is raised in response to different oncogenic stimuli in c*Rb* ^loxP/loxP^ astrocytes. Thus, we observed that Ras induced p38MAPK activation (Figure 4B and Figure S3). Curiously, when the expression of oncogenic Ras is combined with *Rb* deletion the levels of activated p38MAPK are depleted. In the view of ROS levels described above (Figure 3A), we expected similar p38MAPK activation both in c*Rb* ^loxP/loxP^/Ras^V12^ and c*Rb* ^−/−^/Ras^V12^ groups.

The first specific phosphatase of p38MAPK to be identified was Wip1 [50]. Wip1 is a serine/threonine phosphatase of the type 2C protein phosphatase family (PP2C). This family of phosphatases, highly conserved in eukaryotes, is frequently associated with regulation of cellular stress responses [51]. An important amount of data published in the last years suggests that Wip1 is an oncogene. This phosphatase cooperates with other oncogenes to transform murine embryonic fibroblasts [52]. Recently, it has been reported that E2F-1 down-regulates p38MAPK signaling pathway through E2F-induced up-regulation of Wip1 [30]. Accordingly, in our experimental setting *Rb*-null glial cells showed increased levels of this phosphatase, which explains the down-regulation of phosphorylated p38MAPK (Figure 4B).

To further validate our observations, human low- and high-grade glioma sections were labeled for this marker. High levels of Wip1 expression correlate with high-grade tumors (Figure 4C).

**Figure 5:**
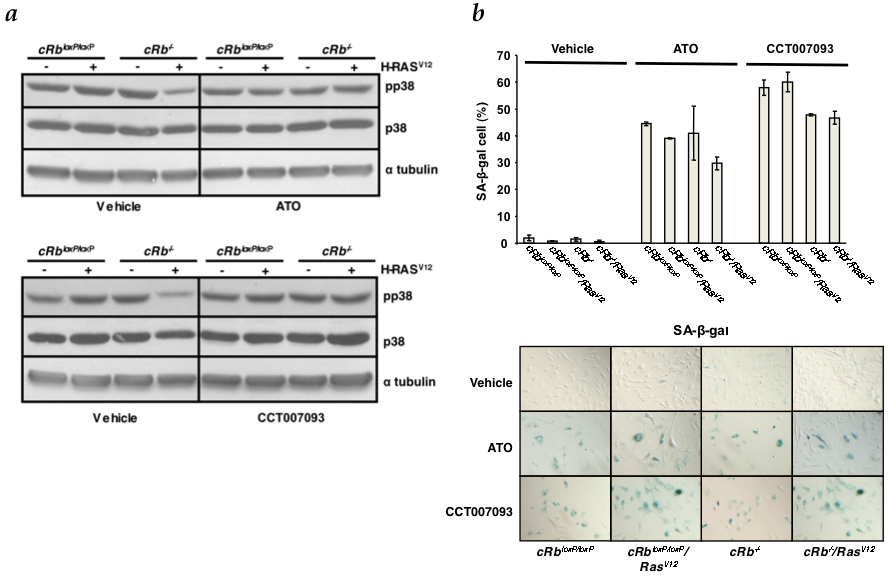
Wip1 inhibition enables senescence in cRb^−/−^/Ras^V12^ astrocytes. a, All the experimental groups were treated with the Wip1 chemical inhibitors CCT007093 and Arsenic Trioxide (ATO). Levels of p-p38MAPK were assessed by Western blot analysis as a readout of Wip1 phosphatase activity inhibition. b, SA-β-galactosidase assays in all groups (without treatment, ATO treatment, CCT007093 treatment). The y-axis represents the percentage of-positive cells (mean and s.d.).

Also, to test our hypothesis regarding the Ras-pRb loss cooperation mediated by Wip1 all experimental groups were treated with two chemical inhibitors upon infection, CCT007093 [57] and Arsenic Trioxide [58]. As a result, dephosphorylation of p38MAPK by Wip1 was blocked as demonstrated by the increased level of phosphorylated p38MAPK, especially in the c*Rb* ^−/−^/Ras^V12^ group (Figure 5A). This biochemical outcome correlates with the positive staining observed in SA-β-Galactosidase assays (Figure 5B). In addition, proliferation rates observed in c*Rb* ^loxP/loxP^/Ras^V12^ and c*Rb* ^−/−^/Ras^V12^ treated groups were significantly lower that the same cells without treatments (Figure S4), confirming previous reports where *Ppm1d* ^−/−^ MEFs showed reduced proliferation rate with features of early senescence [52].

## Discussion

Collectively, our results show that astrocytes expressing oncogenic Ras display a strong proliferation in the absence of senescence, facilitating their transformation into tumoral cells both *in vitro* and *in vivo*. This observation has not been established in other lineages, where OIS blocks tumor progression [17], [48]. In addition to that, the activation of DNA damage checkpoint response detected in these cells raises some questions about the kind of relationship that DDR holds with OIS. Although it has been reported that OIS is a consequence of the activation of DDR [19]–[22] we observed that in astrocytes DDR activation does not necessary lead to senescence. At the same time, the pRb requirement for Ras-mediated transformation [53] was not found in our model. Our data indicate that HRas^V12^ is able to cooperate with *Rb* loss in the tumor malignization. This cooperation between Ras and *Rb* loss opposes the conception that pRb is an essential mediator that links Ras-dependent mitogenic signaling to cell cycle regulation [54].

**Figure 6:**
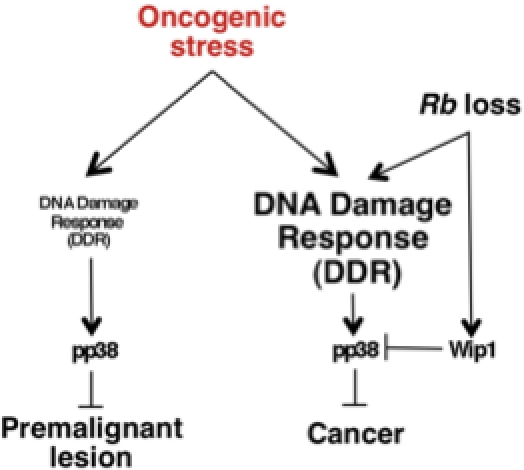
DDR is modulated by *Rb* loss switching premalignant lesion to cancer.

Given that p38MAPK-deficient cells are sensitized to HRas^V12^-induced transformation [25] and although the inactivation of Wip1 is related to the inhibition of tumorigenesis [55], our data point out that the cooperation between Ras and loss of *Rb* could be due to the increase of Wip1 and consequently p38MAPK inactivation. In this way, *Rb* loss in HRas^V12^-expressing astrocytes confers a double advantage. On one hand, *Rb* absence increases the astrocytes proliferative rate but on the other hand, in a *Rb* knock-out context, E2F-1 is activated and up-regulates both DNA damage response in an DNA damage-independent manner, favoring repair mechanisms, and Wip1 [30], leading to an inactivation of p38MAPK and thus increasing cellular transformation. All of this may explain the low latency of c*Rb* ^−/−^/Ras^V12^ tumors versus c*Rb* ^loxP/loxP^/Ras^V12^ tumors and the high Wip1 expression observed in high-grade human gliomas. Interestingly enough, Wip1 chemical inhibition in c*Rb* ^−/−^/Ras^V12^ astrocytes leads to a significant fall in the proliferative rate of these cells, which eventually enter senescence as opposed to cells where Wip1 remains unaffected. Lately, several authors have proposed the use of selective therapeutic inhibitors targeting Wip1 as a promising treatment for different types of cancer [56]–[58]. This study sheds new lights on the possible mechanisms behind the progression from preneoplastic lesions to malignant tumors (Figure 6), and according to our glioma model identifies Wip1 as a potential druggable target for new therapeutic approaches for a tumor whose bad prognosis has not significantly changed over the past two decades.

## Materials and Methods

### Cell culture and SA β-Galactosidase assay

Astrocytes were generated from c*Rb* ^loxP/loxP^ neonatal mice at day 3. The care and use of all experimental animals was in accordance with institutional guidelines. Cells were maintained in Dulbecco modified Eagle medium (Sigma) with 10% fetal bovine serum, 1% L-glutamine, and 1% penicillin/streptomycin (GIBCO-Invitrogen). For introduction of an activated *Ras* allele and *Rb* loss into astrocytes, *Phoenix*-Eco packaging cells (a gift from G.P. Nolan) were transfected with pBABE, pBABE-HRas^V12^, PIG-puro and PIG-CRE retroviral plasmids (a gift from P.P. Pandolfi). SA-β-galactosidase activity was assessed with the Senescence β-Galactosidase Staining Kit (Cell Signaling).

### Immunoblot

Cultures were washed twice in ice-cold PBS, lysed in ice-cold RIPA buffer (1× PBS, 1% Nonidet P-40, 0.5% sodium deoxycholate, 0.1% SDS, 10 mg/ml PMSF, 40 µg of aprotinin/ml, 100 mM orthovanadate). Analysis of protein levels was carried out by immunoblot analysis using polyclonal antibodies (Abs) against p53 (CM5; Novocastra), p-p53 ser15 (9284; Cell Signaling), p19ARF (ab80; Abcam), p38 (C-20; Santa Cruz), p-p38 (Thr180/Tyr182) (sc-17852; Santa Cruz), p16 (M-156; Santa Cruz), p107 (C-18; Santa Cruz), p-H2AX Ser139 (07-164 Upstate) and WIP-1 (H-300; Santa Cruz) and the monoclonal antibodies pan-Ras (Ab-3; Calbiochem), pan-Ras-V^12^ (Ab-1; Calbiochem), anti-α-Tubulin (T5168; Sigma) and Rb (554136; BD). Densitometric analyses were performed using UN-SCAN-IT software (Silk Scientific, UT, USA).

### Immunohistochemistry

Twenty cases of human astrocytomas, ten low-grade (grades I–II) and ten high-grade (grade III–IV), were retrieved from the files of the Pathology Department of the Clinical University Hospital, Santiago de Compostela, Spain. The tumors had been diagnosed according to standard criteria from the last WHO classification. Two tissue microarrays (TMAs) were constructed from these cases using a Tissue Arrayer device (Beecher Instruments, Sun Prairie, WI). Two selected 1.5-mm diameter cylinders from two representative areas histologically reviewed were included in each case. Internal and external appropriate controls were included in each TMA. TMA blocks were sectioned to produce 4-µm sections. Immunohistochemistry was performed using a universal second antibody kit that used a peroxidase-conjugated labeled-dextran polymer (Envision Plus, Dako, Denmark). The following primary antibodies were used: Wip1 (polyclonal, Santa Cruz Biotechnology, USA), phospho-histone H3 (polyclonal, Cell Signaling Technology) and p-p38 (polyclonal, Santa Cruz Biotechnology). Prior to immunostaining, antigen retrieval was performed by the sections in buffer by 20 min (Tris-EDTA pH 9 for Wip1 and p-p38, citrate buffer pH 6 for H3). Overnight incubation at 4°C was performed with every antibody.

### Metaphase chromosome preparation and FISH

Metaphase spreads were prepared from exponentially growing cells after treatment with colcemid (0.1 µg/ml) for 7 hr. Cells were incubated in hypotonic buffer (0.05 M KCl, 0.0034 M trisodium citrate) for 20 min at 37°C and fixed 75% methanol, 25% acetic acid. Cells were then spotted onto microscope slides and stained with 2% Wright in Gurr buffer (pH 7.0). Metaphase chromosomes were scored using a Leica 2005 microscope under a 100× oil objective lens. At least 50 metaphases were analyzed from three independent experiments. TFISH was performed on unstained metaphase chromosomes using a Cy3- labeled peptide nucleic acid probe. For T-FISH, both the DNA probe and the slides were heat denatured (80°C for 5 min) and hybridized at 37°C for 2 hr accordance with the manufacturer's specifications (Dako Cytomation). Slides were counterstained with DAPI, and the images were captured using a Leica 2005 microscope equipped with the software program by Leica 4000.

### Wip1 inhibition treatment

c*Rb* ^loxP/loxP^ conditional astrocytes were co-infected with PIG/pBABE, PIG/pBABE-HRasV12, PIG-Cre/pBABE and PIG-Cre/pBABE-HRas^V12^ retroviral vectors. Cells were plated in triplicate and treated either with CCT007093 (Maybridge, Cornwall, UK) or Arsenic Trioxide (Sigma) in the second day upon infection at a concentration of 25 µM and 10 µM, respectively. Subsequent cell fixation and crystal violet staining were carried out in the indicated days.

## Supporting Information

Figure S1 Telomere fluorescence in situ hybridization (T-FISH) of metaphase spreads. Merged images of DAPI (blue) and telomeric probe (red). The selected regions of metaphase spreads from mouse astrocytes infected with HRASV12 are magnified to show centromere fragments (CF) and Robertsonian centromeric fusion (Rob), indicated by arrows.
(3.00 MB TIF)

Figure S2 Acute deletion of Rb in astrocytes and analysis of pRb and p107 levels. Immunoblot analysis of proliferating cRbloxP/loxP astrocytes after infection at day 1 and 6 post-selection with puromycin. pRb was no longer detectable at day 1 after selection. The pRb family member p107 was significantly affected by loss of pRb. Compensation of pRb loss by p107 presence is already present at day 1.
(3.00 MB TIF)

Figure S3 Acute deletion of Rb in astrocytes and the effect on Wip1 and pp38 levels. Immunoblot analysis of proliferating cRb loxP/loxP astrocytes after infection at day 1 and 6 post-selection with puromycin. The lower level of pp38 is shown after 7 days but not at day 1 after selection.
(3.00 MB TIF)

Figure S4 Wip1 inhibition decreases the proliferation rate in cRb−/−/RasV12 astrocytes. All the experimental groups were treated with the Wip1 chemical inhibitors CCT007093 and Arsenic Trioxide (ATO). The relative cell number of early-passage cRbloxP/loxP conditional astrocytes co-infected with PIG/pBABE (vector), PIG/pBABE-HRasV12, PIG-Cre/pBABE and PIG-Cre/pBABE-HRasV12 retroviral vectors, is shown. After infection, cells were plated in triplicate and fixed on the indicated days for subsequent staining with crystal violet. Each time point represents the mean±s.d. of total cumulative cell number (without treatment, ATO treatment, CCT007093 treatment).
(3.00 MB TIF)
