# Supplementary figures and images for: Retinoblastoma Loss Modulates DNA Damage Response Favoring Tumor Progression
Source: PLoS One. 2008 Nov 5;3(11):e3632. doi: 10.1371/journal.pone.0003632 (PMC2573954; doi:10.1371/journal.pone.0003632)

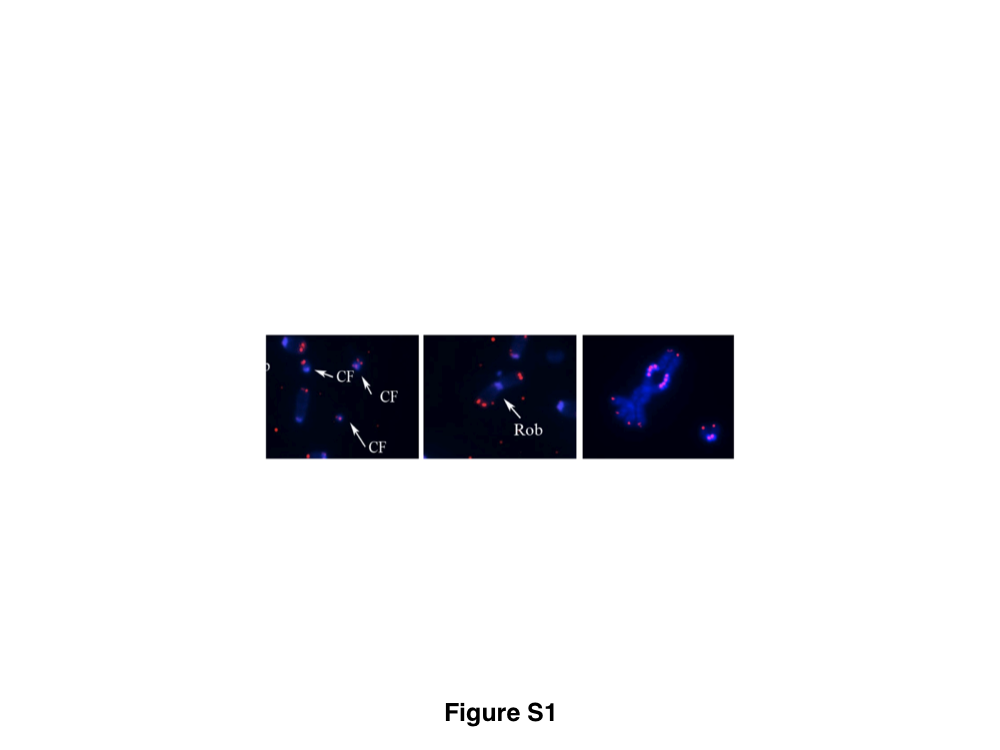

Supplement: Figure S1 — Telomere fluorescence in situ hybridization (T-FISH) of metaphase spreads. Merged images of DAPI (blue) and telomeric probe (red). The selected regions of metaphase spreads from mouse astrocytes infected with HRASV12 are magnified to show centromere fragments (CF) and Robertsonian centromeric fusion (Rob), indicated by arrows. (3.00 MB TIF) [file pone.0003632.s001.tif]

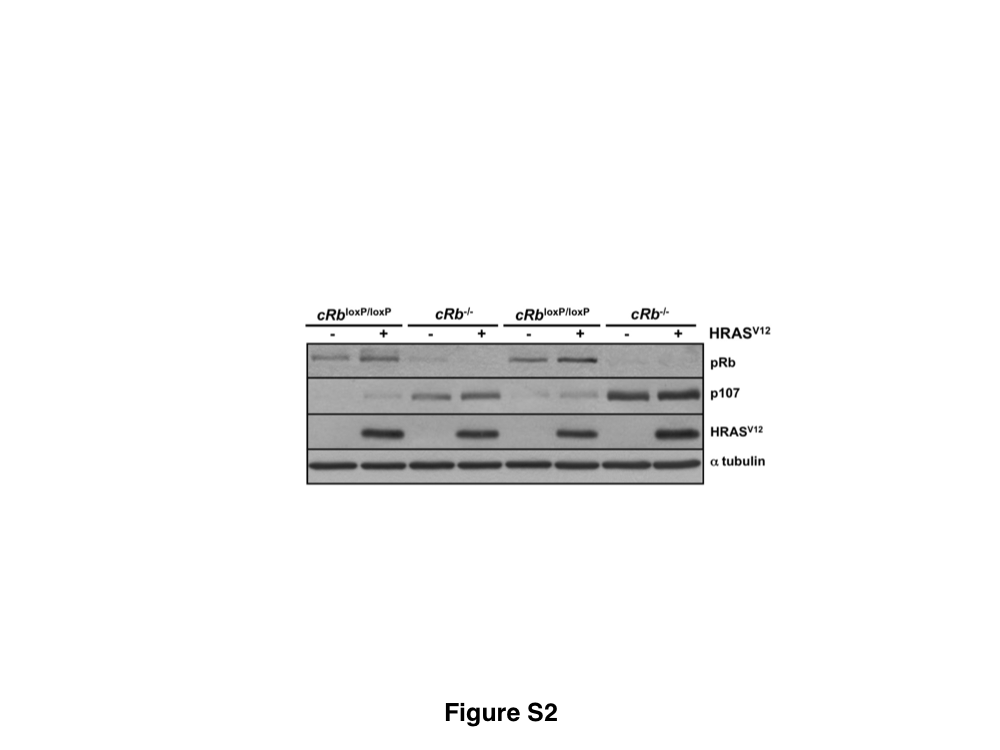

Supplement: Figure S2 — Acute deletion of Rb in astrocytes and analysis of pRb and p107 levels. Immunoblot analysis of proliferating cRbloxP/loxP astrocytes after infection at day 1 and 6 post-selection with puromycin. pRb was no longer detectable at day 1 after selection. The pRb family member p107 was significantly affected by loss of pRb. Compensation of pRb loss by p107 presence is already present at day 1. (3.00 MB TIF) [file pone.0003632.s002.tif]

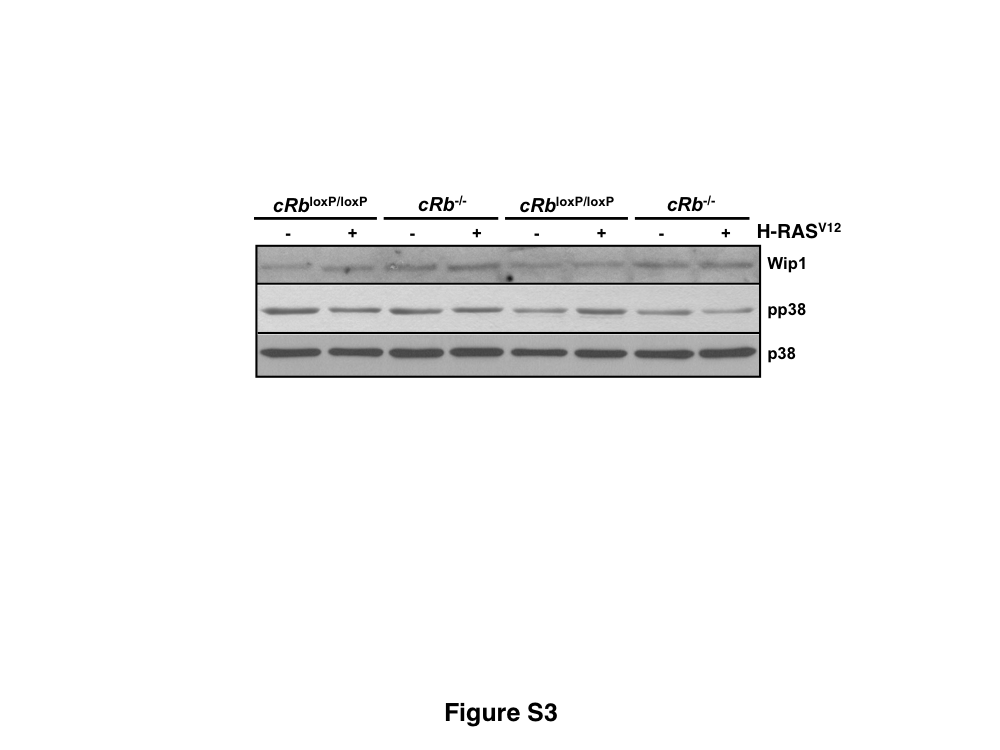

Supplement: Figure S3 — Acute deletion of Rb in astrocytes and the effect on Wip1 and pp38 levels. Immunoblot analysis of proliferating cRb loxP/loxP astrocytes after infection at day 1 and 6 post-selection with puromycin. The lower level of pp38 is shown after 7 days but not at day 1 after selection. (3.00 MB TIF) [file pone.0003632.s003.tif]

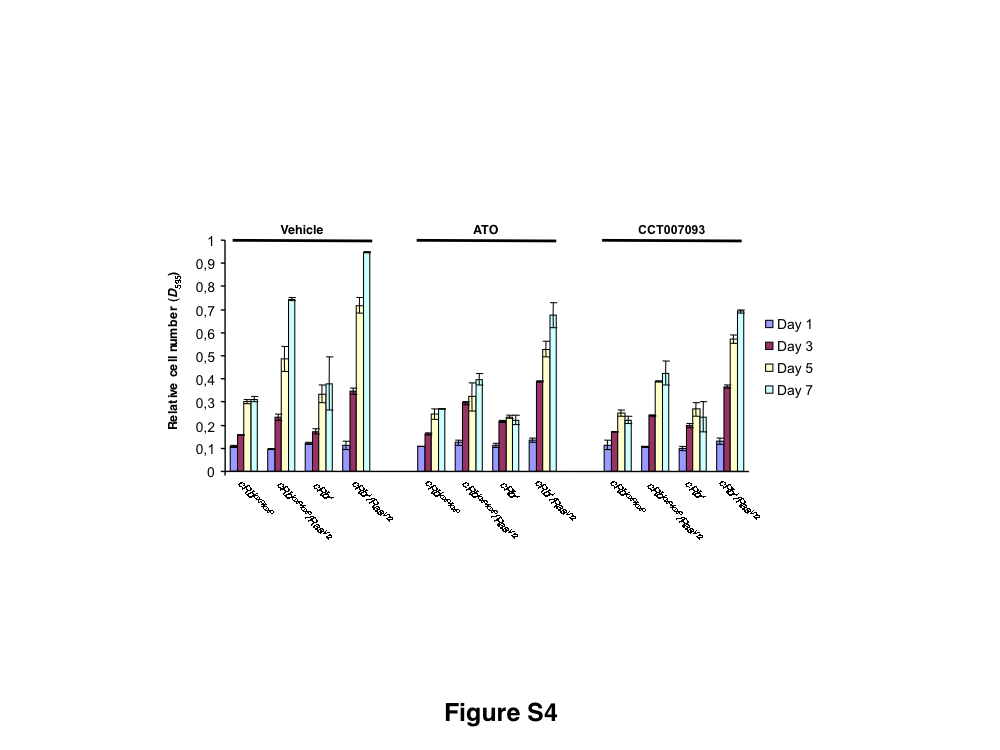

Supplement: Figure S4 — Wip1 inhibition decreases the proliferation rate in cRb−/−/RasV12 astrocytes. All the experimental groups were treated with the Wip1 chemical inhibitors CCT007093 and Arsenic Trioxide (ATO). The relative cell number of early-passage cRbloxP/loxP conditional astrocytes co-infected with PIG/pBABE (vector), PIG/pBABE-HRasV12, PIG-Cre/pBABE and PIG-Cre/pBABE-HRasV12 retroviral vectors, is shown. After infection, cells were plated in triplicate and fixed on the indicated days for subsequent staining with crystal violet. Each time point represents the mean±s.d. of total cumulative cell number (without treatment, ATO treatment, CCT007093 treatment). (3.00 MB TIF) [file pone.0003632.s004.tif]
